# Improving the efficacy for meropenem therapy requires a high probability of target attainment in critically ill infants and children

**DOI:** 10.3389/fphar.2022.961863

**Published:** 2022-10-05

**Authors:** Zeming Wang, Jing Bi, Dianping You, Yu Tang, Gang Liu, Jinqian Yu, Zhipeng Jin, Tingting Jiang, Xue Tian, Hui Qi, Lei Dong, Lili Dong, Qunqun Zhang, Wei Zhao, Adong Shen

**Affiliations:** ^1^ Beijing Key Laboratory of Pediatric Respiratory Infection Diseases, Key Laboratory of Major Diseases in Children, Ministry of Education, National Clinical Research Center for Respiratory Diseases, National Key Discipline of Pediatrics (Capital Medical University), Beijing Pediatric Research Institute, Beijing Children’s Hospital, Capital Medical University, National Center for Children’s Health, Beijing, China; ^2^ Department of Pediatrics, Beijing Friendship Hospital, Capital Medical University, Beijing, China; ^3^ Baoding Children’s Hospital, Baoding, China; ^4^ Children’s Hospital of Hebei Province, Shijiazhuang, China; ^5^ Children’s Hospital Affiliated to Zhengzhou University, Henan Children’s Hospital, Zhengzhou Children’s Hospital, Zhengzhou, China; ^6^ Department of Infection Diseases, Beijing Children’s Hospital, Capital Medical University, National Center for Children’s Health, Beijing, China; ^7^ Department of Neonatology, Sunyi Women’s and Children’s Hospital of Beijing Children’s Hospital, Beijing, China; ^8^ Department of Clinical Pharmacy, School of Pharmaceutical Sciences, Cheeloo College of Medicine, Shandong University, Jinan, China; ^9^ NMPA Key Laboratory for Clinical Research and Evaluation of Innovative Drug, Qilu Hospital of Shandong University, Shandong University, Jinan, China

**Keywords:** fT_>MIC_, meropenem, children, critically ill, pharmacodynamic target attainment

## Abstract

Probability of target attainment is the key factor influencing the outcome of meropenem therapy. The objective of the present study was to evaluate the relationship between the time in which the plasma free concentration of meropenem exceeds the minimum inhibitory concentration of pathogens (*fT*_>MIC_) during therapy and the clinical outcome of treatment to optimize meropenem therapy. Critically ill children with infections who had received intravenous meropenem monotherapy were included. The relationship between *fT*_>MIC_ of meropenem and effectiveness and safety were explored. Data from 53 children (mean age ± standard deviation, 26 months ± 38) were available for final analysis. Children with *fT*_>MIC_ ≥ 5.6 h (*n* = 14) had a more significant improvement in antibacterial efficacy in terms of decrease in fever (*p* = 0.02), white blood cell count (*p* = 0.014), and C-reactive protein (*p* = 0.02) compared with children with *fT*_>MIC_ < 5.6 h (*n* = 39) after meropenem therapy completed. No drug-related adverse events were shown to have a causal association with meropenem therapy. Our study shows the clinical benefits of sufficient target attainment of meropenem therapy. Meeting a suitable pharmacodynamic target attainment of meropenem is required to ensure better antibacterial efficacy in critically ill infants and children.

**Clinical Trial Registration:** clinicaltrials.gov, Identifier NCT03643497.

## Introduction

Meropenem is the most widely used carbapenem in children for the treatment of severe infections owing to its broad antimicrobial spectrum (including multidrug-resistant bacteria) and favorable safety profile (Thalhammer and Horl, 2000; Cies et al., 2014). Meropenem shows time-dependent antibacterial activity related to the time that the plasma free concentration of meropenem exceeds the minimum inhibitory concentration (MIC) of the pathogen (*fT*_>MIC_) (Mathew et al., 2016). Despite its wide use, the standard dosing regimen, administered as 10–40 mg/kg/dose (q8h) infused for 0.5 h, for critically ill infants, children (Kongthavonsakul et al., 2016; Cies et al., 2017; Hassan et al., 2019; Wang et al., 2020) and adults(Alsultan et al., 2021) may fail to meet pharmacodynamic (PD) targets.

Probability of target attainment (PTA) is the key factor influencing the outcome of meropenem therapy. A higher target *fT*_>MIC_ value ≥70% of the dosage interval has been suggested for patients with severe bacterial infections to ensure bactericidal effectiveness, however, limited data are available in children to support this target (Wang et al., 2020, Cies et al., 2017). Additionally, for some critically ill children without renal impairment, clearance (CL) of therapeutics is augmented thereby decreasing plasma levels, and the volume of distribution (V_d_) is increased due to a hyper dynamic state in response to inflammation (Wang et al., 2020; Cies et al., 2017; Rapp et al., 2019). As a result, the probability of target attainment (PTA) of 70% *fT*_>MIC_ with a standard meropenem dosing regimen is low in critically ill children (Wang et al., 2020). Failure to combat the causative pathogen adequately as a result of a low PTA clearly reduces the probability of successful treatment.

We hypothesized that PTA is the key factor for meropenem treatment success in critically ill children. To optimize meropenem therapy, the relationship between 70% *fT*_>MIC_ of meropenem [70% × 8 h (the dosage interval) = 5.6 h] and clinical outcome was evaluated in this study.

## Methods

### Study design

A multicenter prospective, open-label PD study of meropenem was conducted in Beijing Children’s Hospital, Baoding Children’s Hospital, Henan Children’s Hospital, Hebei Children’s Hospital from 2019 to 2021. Children hospitalized in pediatric intensive care units with bacterial meningitis [National Institute for Health and Care Excellence (NICE), 2018], sepsis (Dellinger et al., 2013) or severe pneumonia (Subspecialty Group of Respiratory Diseases, 2013) who had received meropenem monotherapy (Dainippon Sumitomo Pharma, Osaka, Japan) for a clinically suspected or proven bacterial infection as an intravenous infusion for 0.5–1 h at 20–40 mg/kg/dose (q8h) were included. Exclusion criteria included a history or evidence of chronic pathology of any organ system, receiving meropenem for ≤48 h, non-bacterial infections, and incomplete clinical information. Demographic data, clinical “characteristics,” and response to/adverse events (AE) associated with meropenem therapy were recorded in H6WORLD system (https://www.h6world.cn/home) for the management of clinical data independently.

### Calculation of *fT*_>MIC_ for meropenem

Opportunistic PK samples were collected and plasma concentrations of meropenem were quantified using high-performance liquid chromatography (HPLC) as described in our previous study (Wang et al., 2020). On the basis of the population pharmacokinetic (PK)-PD parameters of meropenem in critically ill infants and children with infections reported (Wang et al., 2020), the *fT*_>MIC_ for each patient was calculated using the following equation (Dudley and Ambrose, 2000; Bradley et al., 2008; Yun et al., 2010):

$$\frac{In\frac{Dose}{Vd}-In\;MIC}{\frac{0.693}{T_{1/2}}}$$ (1)

where *V*_d_ is the volume of distribution, *T*_1/2_ is the serum elimination half-life, and Dose is the single dose administered. The information associated with *V*_d_ and *T*_1/2_ value is detailed in our article (Wang et al., 2020). Meropenem has excellent activity against Gram-positive bacteria, including *Streptococcus pneumoniae*, and Gram-negative organisms such as *Escherichia coli*, *Haemophilus influenzae*, *Klebsiella pneumonia*, and *Neisseria meningitidis*; these were the most common bacterial pathogens observed in our study. The MICs for almost all pathogens were ≤1 mg/L (CLSI supplement M100. Wayne, 2019) and the PTA was 18.7% (MIC = 1 mg/L) and 5.8% (MIC = 2 mg/L) at the standard dosing regimen for meropenem under 70% *fT*_>MIC_ based on our microbiological data (Wang et al., 2020). In order to ensure enough children in the PD target attainment group for efficacy analysis, MIC = 1 mg/L with higher PTA was selected. It should be highlighted that the choice of the 5.6-h cut-off value for meropenem was based on a high PTA at 70% *fT*_>MIC_ (8-h dose interval). The 70% *fT*_>MIC_ (defined as the time that the plasma free concentration of meropenem exceeds the MIC) value of meropenem was calculated as 70% × 8 h (the dosage interval) = 5.6 h. Patients with *fT*_>MIC_ values ≥ 5.6 h were identified as achieving *fT*_>MIC_ of at least 70% and meeting the target attainment. The relationship between meropenem *fT*_>MIC_ and effectiveness and safety were explored.

### Effectiveness assessment

The assessment of response to meropenem therapy was based on clinical characteristics (signs and symptoms of infection and inflammatory markers) and/or radiological (X-ray or computed tomography scans) findings at first 48–72 h after meropenem treatment and the point when meropenem therapy was completed. Clinical response was defined as improvements in signs and symptoms, laboratory testing values, and infection resolution without worsening of severe pneumonia by chest X-ray examination. Failure was defined as the demonstration of no improvement or deterioration of signs and symptoms, laboratory testing values, or the requirement of additional antibiotics (Schuler, 1995). The effective rates (ER) were calculated using the following equation:

$$ER=\frac{Ni}{N}\times 100\%$$ (2)

where Ni is the numbers of patients with clinical improvement in children with *fT*_>MIC_ ≥ 5.6 h or children with *fT*_>MIC_ < 5.6 h, N is the total numbers of children in the corresponding group above.

### Safety assessment

AEs that occurred during meropenem use were recorded and classified by severity (mild, moderate, severe, or life-threatening) and relationship (possibly, probably, certainly, probably not, or certainly not related) to treatment by the investigator independently before and after therapy (Cohen-Wolkowiez et al., 2012; Liu et al., 2018).

### Statistical analysis

Data are expressed as the means ± standard deviation (SD). Proportions were compared by the ***χ*** ^2^ or Fisher’s exact tests, as appropriate. Continuous variables were compared by the *t*-test or Wilcoxon test, as appropriate. Statistical significance was defined by a two-sided *p* value of 0.05. All statistical analyses were performed using IBM SPSS software version 25.0.

## Results

### Patients

From July 2019 to November 2021, 98 critically ill children with infections who had received intravenous meropenem were screened. There were 53 patients (18 bacterial meningitis, 24 sepsis, and 11 severe pneumonia patients) available for PD analysis after exclusion of 35 patients on two antibiotics (including meropenem), one patient with Kawasaki disease, three patients out of study, three patients with trauma, two patients who had been infected with virus combined and one patient with meropenem treatment for 1 day. The mean age and weight of the 53 children at the time of study were 26 ± 38 months and 12.67 ± 11.07 kg, respectively. There were no significant differences in diseases distribution between the two groups (Table 1).

**TABLE 1:** Demographic parameters.

Variable	Meropenem T_>MIC_ (hour)		*p*
	≥5.6 h (*n* = 14)	<5.6 h (*n* = 39)	
Demographic data			
Age (months), mean (SD)	14 (24)	30 (41)	0.194
Boys, n (%)	10 (71.43)	21 (53.85)	0.252
Weight (kg), mean (SD)	8.97 (5.15)	14.00 (12.26)	0.151
Creatinine clearance (ml/min/1.73 m^2^), mean (SD)	129.56 (75.66)	177.26 (66.09)	***0.03***
Severe infectious diseases *n* (%)			
Sepsis	8 (57.14)	16 (41.03)	0.299
Bacterial meningitis	4 (28.57)	14 (35.90)	0.620
Severe pneumonia	2 (14.29)	9 (23.08)	0.487
Pathogens, *n* (%)			
*Klebsiella pneumoniae*	0 (0.00)	3 (7.69)	
*Staphylococcus aureus*	1 (7.14)	2 (5.13)	
*Listeria monocytogenes*	0 (0.00)	1 (2.56)	
*Pseudomonas aeruginosa*	2 (14.29)	0 (0.00)	
*Streptococcus pneumoniae*	1 (7.14)	1 (2.56)	
*Escherichia coli*	1 (7.14)	0 (0.00)	
*Haemophilus influenzae*	0 (0.00)	1 (2.56)	
*Acinetobacter baumannii*	1(7.14)	0 (0.00)	
*Viridans streptococci*	1(7.14)	0 (0.00)	
*Streptococcus constellatus*	0 (0.00)	1 (2.56)	
No pathogens found	8 (57.14)	30 (76.92)	
Duration of antibiotic treatment (days), mean (SD)	11.79 (7.24)	13.18 (7.82)	0.57
Duration of hospitalization (days), mean (SD)	15.64 (10.35)	19.74 (16.01)	0.378

The bold and Italics indicated that the *p* value was less than 0.05. The difference was statistically between children with fT>MIC ≥ 5.6 h and children with fT>MIC < 5.6 h.

### Calculation of meropenem *fT*_>MIC_ levels

Meropenem was administrate in the enrolled patients with sepsis, severe pneumonia (20 mg/kg/dose) and bacterial meningitis (40 mg/kg/dose) respectively. The median duration of therapy was 13 days. 111 meropenem concentrations were obtainable. The median number of samples per patients was 2 (range, 1–4). The median meropenem concentration of PK samples was 1.16 (range, 0.2–147.24) μg/ml. The median *fT*_>MIC_ was 4.38 h (range, 3.48–8.0 h).

### Effectiveness assessment

*fT*_>MIC_ of meropenem was <5.6 h in 39 cases (73.58%) and ≥5.6 h in 14 cases (26.42%). Significant improvement in antibacterial efficacy at first 48–72 h after meropenem treatment (ER: 78.57% v 35.90%, *p* = 0.006) and the point when meropenem therapy was completed (ER: 92.86% v 64.10%, *p* = 0.04) was found in children with *fT*_>MIC_ ≥ 5.6 h compared with children with *fT*_>MIC_ < 5.6 h. Significantly decreases in fever were present between the two groups at first 48–72 h (*p* = 0.002) and the point meropenem therapy completed (*p* = 0.02). Additionally, Clear decreases in white blood cell (WBC) counts (*p* = 0.014) and C-reactive protein (CRP) (*p* = 0.02) were also observed between the two groups after meropenem treatment. No significant differences were observed for other effectiveness parameters (Table 2).

**TABLE 2:** Clinical response to meropenem therapy in children.

Response	Meropenem T_>MIC_ (hour)		*p*
	≥5.6 h (*n* = 14)	<5.6 h (*n* = 39)	
Total patients, *n* (%)			
Improvement			
Meropenem treatment after first 48–72 h	11 (78.57)	14 (35.90)	***0.006***
Meropenem treatment completed	13 (92.86)	25 (64.10)	***0.04***
Failure			
Meropenem treatment after first 48–72 h	3 (21.43)	25 (64.10)	***0.006***
Meropenem treatment completed	1 (7.14)	14 (35.90)	***0.04***
Fever, patients *n* (%)			
Improvement			
Meropenem treatment after first 48–72 h	13 (92.86)	18 (46.15)	***0.002***
Meropenem treatment completed	13 (92.86)	23 (58.97)	***0.02***
Failure			
Meropenem treatment after first 48–72 h	1 (7.14)	21 (53.85)	***0.002***
Meropenem treatment completed	1 (7.14)	16 (41.03)	***0.02***
WBC, patients *n* (%)			
Meropenem treatment after first 48–72 h	10 (71.43)	27 (69.23)	0.878
Meropenem treatment completed	13 (92.86)^#^	22 (56.41)^#^	***0.014***
CRP, patients *n* (%)			
Meropenem treatment after first 48–72 h	13 (92.86)	26 (66.67)	0.057
Meropenem treatment completed	13 (92.86)*	23 (58.97)*	***0.02***

^#^ White blood cell (WBC) count decreased under 10 × 10^9^/L; * C-reactive protein (CRP) decreased under 10 mg/L; *p* represents statistical significance between children with *fT*_>MIC_ ≥ 5.6 h and children with *fT*_>MIC_ < 5.6 h.

The bold and Italics indicated that the *p* value was less than 0.05. The difference was statistically between children with fT>MIC ≥ 5.6 h and children with fT>MIC < 5.6 h.

*fT*_>MIC_ of meropenem was <5.6 h in 14 cases (77.78%) and ≥5.6 h in 4 cases (22.22%) in children with bacterial meningitis. More decreases in fever, WBC and CRP were found between the two groups at first 48–72 h and the point meropenem therapy completed. However, only improvement in fever was significant at the early treatment stage(*p* = 0.023) (Table 3).

**TABLE 3:** Clinical response to meropenem therapy in children with bacterial meningitis or spesis and severe pneumonia.

Response	Meropenem T_>MIC_ (hour)		*p*	Meropenem T_>MIC_ (hour)		*p*
	≥5.6 h (*n* = 4)	<5.6 h (*n* = 14)		≥5.6 h (*n* = 10)	<5.6 h (*n* = 25)	
Total patients, *n* (%)	Bacterial meningitis			Spesis and severe pneumonia		
Improvement						
Meropenem treatment after first 48–72 h	3 (75.00)	5 (35.71)	0.163	8 (80.00)	9 (36.00)	***0.019***
Meropenem treatment completed	4 (100.00)	7 (50.00)	0.07	9 (90.00)	18 (72.00)	0.252
Fever, patients *n* (%)						
Improvement						
Meropenem treatment after first 48–72 h	4 (100.00)	5 (35.71)	***0.023***	9 (90.00)	13 (52.00)	***0.036***
Meropenem treatment completed	4 (100.00)	7 (50.00)	0.07	9 (90.00)	16 (64.00)	0.124
WBC, patients *n* (%)						
Meropenem treatment after first 48–72 h	3 (75.00)	9 (64.29)	0.688	7 (70.00)	18 (72.00)	0.906
Meropenem treatment completed	4 (100.00)^#^	8 (57.14)^#^	0.109	9 (90.00)^#^	14 (56.00)^#^	0.056
CRP, patients *n* (%)						
Meropenem treatment after first 48–72 h	4 (100.00)	8 (57.14)	0.109	9 (90.00)	18 (72.00)	0.252
Meropenem treatment completed	4 (100.00)*	8 (57.14)*	0.109	9 (90.00)*	15 (60.00)*	0.084

^#^ White blood cell (WBC) count decreased under 10 × 10^9^/L; * C-reactive protein (CRP) decreased under 10 mg/L; *p* represents statistical significance between children with *fT*_>MIC_ ≥ 5.6 h and children with *fT*_>MIC_ < 5.6 h.

The bold and Italics indicated that the *p* value was less than 0.05. The difference was statistically between children with fT>MIC ≥ 5.6 h and children with fT>MIC < 5.6 h.

### Safety assessment

All 53 patients were assessed for treatment safety. Fourteen mild AEs were reported in ten (18.87%) patients, including granulocytopenia (6), aspartate aminotransferase (5), alanine aminotransferase increase (2), and rash (1). There was no statistically significant difference in meropenem *fT*_>MIC_ between children with or without AEs (*p* = 0.715). No patients discontinued meropenem treatment in response to AEs and no drug-related AEs were causally associated with meropenem treatment.

## Discussion

Optimizing PK exposure of antibiotics to meet suitable target attainment in critically ill patients could improve infection-related outcomes and reduce bacterial antibiotic resistance. A previous study reported that adjusting doses to achieve a *fT*_>MIC_ of at least 70% of the dosage interval was more likely to eradicate the causative pathogen (Scaglione et al., 2009). To the best of our knowledge, our study shows the clinical benefits of a high PTA for meropenem therapy in critically ill children.

To ensure better clinical outcome, 70% *fT*_>MIC_ was selected as the PD target in critically ill infants and children associated with immunodeficient states (Ariano et al., 2005; Scaglione et al., 2009; Franciscus van der Meer et al., 2011). Our study did find that there was significantly clinical improvement in children with *fT*_>MIC_ ≥ 5.6 h than children with *fT*_>MIC_ < 5.6 h. We further simulated the clinical efficacy of meropenem with lower *fT*_>MIC_ (60% of the dosage interval = 4.8 h) as the PD target in these children. However, it was no significant difference between the children with *fT*_>MIC_ ≥ 4.8 h and children with *fT*_>MIC_ < 4.8 h (*p* > 0.05, range 0.141–0.905).

According to the instructions, meropenem was administered at 40 or 20 mg/kg in children with bacterial meningitis or other infections, respectively. There was no difference in disease distribution between two groups (*p* = 0.299–0.620). Therefore minor effect under different doses for meropenem was related with our conclusion. In addition, our study showed the better clinical benefits (fever, WBC and CRP) of a high PTA of meropenem therapy in children with bacterial meningitis (*n* = 18). Unfortunately, most of the differences were not significant which associated with the limited patients enrolled. Similar results were also found in children (*n* = 35) with severe pneumonia (*n* = 11) and sepsis (*n* = 24) (Table 3). When the analysis was performed target the whole patients, the differences between the two groups were dramatical as the total population increased (Table 2).

In our study, clinical signs improved in 71.70% cases, which is similar to what Punpanich et al. (2012) reported (68.1%) with a standard dosing regimen for meropenem. However, their regimen could not reach the PD target for susceptible and multi-resistant organisms in critically ill children (Wang et al., 2020). As shown here, optimization of the dose regimen for meropenem is a viable means of improving its efficacy. Children with a high PTA had a significant improvement in antibacterial efficacy compared with children with low target attainment at both the early and final stages of meropenem treatment. Length of hospitalization also decreased in children with a high PTA, although the difference between the two groups was not statistically significant, which is possibly attributed to the limited number of patients. Furthermore, the proportion of patients with decreased inflammatory markers in the group with *fT*_>MIC_ ≥ 5.6 h was lower than that in the group with *fT*_>MIC_ < 5.6 h, but this was also not significant. However, for the patients with inflammatory markers reduced to normal levels, there were significant differences between the two groups. Taken together, a high PTA observably increases the probability of recovery.

We further found that the children with *fT*_>MIC_ < 5.6 h had higher creatinine clearance than children with *fT*_>MIC_ ≥ 5.6 h (177.26 v129.56 ml/min/1.73 m^2^), which resulted in rapid excretion of meropenem and lower *fT*_>MIC_. To determine the optimal dosage regimens, we evaluated treatment frequency and infusion time, showing that 40 mg/kg/dose (q8h) with 4-h infusion and 110 mg/kg/day with continuous infusion can achieve PTA for bacteria with a low MIC (≤2 mg/L) and high MIC (2–8 mg/L), respectively (Wang et al., 2020). Multicenter random clinical trials associated with the efficacy and safety of these recommended regimens are under way.

There are several limitations in our study. Firstly, this study was limited by the small number of children enrolled. Secondly, the selection of the cut-off value (70% *fT*_>MIC_ = 5.6 h, MIC = 1 mg/L) for PTA of meropenem was mainly based on our microbiological and PK-PD data. Different values and MICs might be targeted with bacterial pathogens with higher MICs. Finally, only patients on meropenem monotherapy were included in this study. Because combined antimicrobial therapy might introduce additional complicating factors in the interpretation of the relationship between PTA of meropenem and clinical outcome.

## Conclusion

Our study demonstrates the clinical benefits of a high PTA with meropenem therapy in critically ill children with infections. Children with meropenem *fT*_>MIC_ ≥ 5.6 h showed better antibacterial efficacy. Prospective trials to confirm this discovery in a larger pediatric population will be conducted in future studies.

## Data Availability

The original contributions presented in the study are included in the article, further inquiries can be directed to the corresponding authors.
